# The Multifaceted Role of HSF1 in Pathophysiology: Focus on Its Interplay with TG2

**DOI:** 10.3390/ijms22126366

**Published:** 2021-06-14

**Authors:** Luca Occhigrossi, Manuela D’Eletto, Nickolai Barlev, Federica Rossin

**Affiliations:** 1 Department of Biology, University of Rome ‘Tor Vergata’, 00133 Rome, Italy; luc.occhigrossi@gmail.com (L.O.); manuela.deletto@gmail.com (M.D.); 2 Institute of Cytology, 194064 Saint-Petersburg, Russia; nick.a.barlev@gmail.com; 3 Moscow Institute of Physics and Technology (MIPT), 141701 Dolgoprudny, Russia

**Keywords:** HSF1, heat shock proteins, Transglutaminase 2, diseases, development

## Abstract

The cellular environment needs to be strongly regulated and the maintenance of protein homeostasis is crucial for cell function and survival. HSF1 is the main regulator of the heat shock response (HSR), the master pathway required to maintain proteostasis, as involved in the expression of the heat shock proteins (HSPs). HSF1 plays numerous physiological functions; however, the main role concerns the modulation of HSPs synthesis in response to stress. Alterations in HSF1 function impact protein homeostasis and are strongly linked to diseases, such as neurodegenerative disorders, metabolic diseases, and different types of cancers. In this context, type 2 Transglutaminase (TG2), a ubiquitous enzyme activated during stress condition has been shown to promote HSF1 activation. HSF1-TG2 axis regulates the HSR and its function is evolutionary conserved and implicated in pathological conditions. In this review, we discuss the role of HSF1 in the maintenance of proteostasis with regard to the HSF1-TG2 axis and we dissect the stress response pathways implicated in physiological and pathological conditions.

## 1. Introduction

Protein homeostasis (or proteostasis) could be defined as a network of molecular interactions able to maintain the balance of the whole proteome. The main processes that affect proteostasis include biosynthesis, folding, assembly/disassembly and degradation of proteins and they are interconnected with each other, by forming a dynamic system [1]. The key modulators of proteostasis are known as molecular chaperones, a functional class of proteins able to facilitate polypeptide folding, avoiding the misfolding and thus an improper aggregation between different proteins [2]. Many cellular signaling regulate proteostasis in order to buffer a deleterious accumulation of misfolded proteins and, among these, the heat shock response (HSR) is known as the king pathway to mediate chaperone expression [3]. The HSR activation allows the cells to increase the expression of genes involved in the proteotoxic stress protection and to trigger a signaling to promote cellular rest [4,5,6]. In fact, different factors induce stress by affecting the redox state of the cells that turn out in an increased level of misfolded peptides, which in turn can be detrimental due to their activity alteration [7]. To fight this stress, cells induce synthesis of highly conserved proteins, termed Heat Shock Proteins (HSPs), molecular chaperones that protect cells from harmful stimuli. HSPs, by helping the folding of damaged proteins or driving them to degradation, attempt to avoid their dangerous accumulation. Instead, in physiological conditions, HSPs are involved in the conformational change of peptides, helping assembly and disassembly of multiproteic complex [8]. According to their molecular weight, HSPs are classified in five classes, including the Hsp90, Hsp70, Hsp60, Hsp40 and “small heat shock proteins”. Members of these classes can be constitutively expressed or induced by stress and their intracellular distribution depends on the specific function, for example, localizing in the cytosol as well as in the nucleus (Hsp27, Hsc70, Hsp70 and Hsp90), in the endoplasmic reticulum (Grp78 and Grp94) and in the mitochondrial space (Grp75, Hsp60, TRAP1) [9,10,11]. HSPs show a common molecular structure, with the exception of the small HSPs, containing an ATP binding N- terminal domain (“Nucleotide Binding Domain, NBD”), a C-terminal domain able to bind the peptide, (“Peptide/Substrate Binding Domain”, PBD/SBD) and an interdomain region that facilitate the ATPase activity of the chaperones and the recognition of the substrate [12,13,14]. The bond of the peptides to the PBD domain occurs thanks to the aminoacidic carboxyterminal sequence EEDV (Glu-Glu-Asp-Val) of the chaperones regulated both at the intramolecular level, through hydrolysis and release of the nucleotide in the NBD domain, as well as at the intermolecular level via the activity of several cofactors [15,16]. Co-chaperones and nucleotide exchanging factors (NEFs) regulate the substrate affinity and the ATPasic cycle of the main chaperones. For instance, as Hsp70 interacts with their substrates thanks to co-chaperones DnaJ (Hsp40), which drives the misfolded or unfolded proteins toward chaperones and releasing them upon the association of cofactors NEFs [17,18]. Considering the main role of HSF1 in the regulation of the HSR and the recent studies showing the interaction with type 2 Transglutaminase (TG2), in the following paragraphs we will discuss the importance of the HSF1-TG2 axis in the regulation of the HSR and potential involvements in pathological conditions.

## 2. HSF1: The Master Regulator of the HSR

The heat shock response is mediated by several transcription factors named “heat shock factors” (HSFs), which can bind specific DNA sequences, the heat shock elements (HSEs), located upstream in the promoters of heat shock genes [19,20]. The promoters of the target genes contain several HSEs sequences, thus allowing a simultaneous binding of many HSFs. Moreover, the association of an HSF protein with the HSEs occurs in a cooperative manner, where the binding of an HSF protein facilitates that of the next factor [21]. The HSFs contain several functional domains including a DNA-Binding Domain (DBD), two N-terminal oligomerization domains (“heptad repeat A/B”, HR-A/B), an oligomerization domain (HR-C), a C-terminal activation domain (AD) and a regulatory domain [22,23,24]. In vertebrates, four main transcription factors (HSF1-4) have been identified, but HSF1 shows the most prominent role in the regulation of HSR and HSPs expression [25,26,27]. The sequencing of the HSF1 gene highlighted the fact that the codifying DBD and HR-A/B exons were conserved among the orthologous genes, while several mutations were accumulated on the HR-C and AD domains [28]. HSF1 plays numerous physiological functions during cellular growth and differentiation, it regulates key genes for energy production and it is involved in the cytoskeletal organization [29,30]. However, the main function of HSF1 concerns the modulation of HSPs synthesis in response to stress, since HSF1 knock-out cells do not develop thermo-tolerance making them more sensitive to stress-induced apoptosis [31,32]. HSF1 activation is mediated by a series of regulatory events. In the physiological condition, HSF1 is present in the cytoplasm as inactive monomer, because of the intramolecular binding between HR-C and HR-A/B domains and its association to Hsp90 and Hsp70 [33,34,35]. On the contrary, in a stress condition, HSF1 is released by the inhibitory proteins and subsequently moves into the nucleus binding the HSEs on the promoters of the target genes [36,37]. The HSF1-mediated transcription activation is regulated by a series of phosphorylations on different sites: the phosphorylation on serine 230 and 326 stimulates the transcriptional activity of HSF1, while the phosphorylation on serine 419 and 320, respectively, modulates the nuclear translocation and the DNA binding capability of the transcription factor [37,38]. In addition to the activating phosphorylation, inhibitory phosphorylation also takes place on HSF1 and occurs on serine 121, 303, 307 and 363, to repress the HSF1 activity [37,39,40]. Moreover, HSF1 activation requires the transition from the monomeric to the trimeric form of the transcription factor. Specifically, two cysteine residues (cys35 and 105), localized in the DBD domain, are essential for the formation of disulfide bridges in the trimeric HSF1 [41]. Recently, it has been shown that HSF1 trimerization is not a spontaneous event that occurs following proteotoxic stimuli, but it is mediated by the type 2 Transglutaminase (TG2), a ubiquitous enzyme activated during stress condition [42]. Moreover, a persistent stress leads to an inactivation of HSF1 trough both additional post-translational modifications and a negative feedback mechanism by which the transcription factor is bound by the newly synthetized HSPs [43]. In fact, the HSF1 trimers interact with newly produced Hsp70 and Hsp40 and this binding compromises the transcription factor activity [44].

### 2.1. TG2-Dependent Activation of HSF1

**Figure 1:**
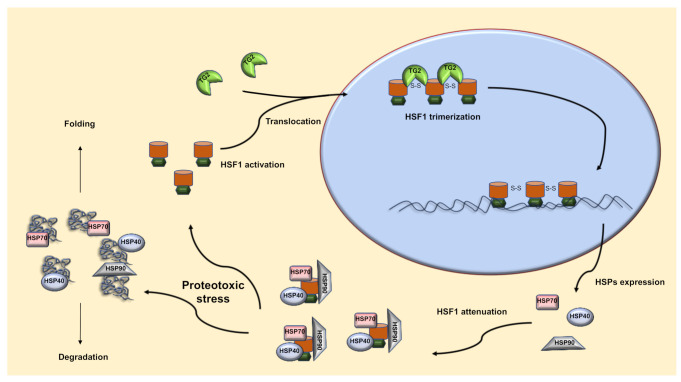
The Heat Shock Response. A proteotoxic stress leads to an increase in misfolded proteins that are bound by HSPs to mediate proteins folding or their degradation. Activated HSF1 moves into the nucleus where it is trimerized by TG2. HSF1 trimers bind HSE sequences stimulating HSPs expression in order to reduce the misfolded peptides accumulation. The new synthetized chaperones interact with monomeric HSF1 to attenuate the heat shock response.

“Tissue” or type 2 Transglutaminase (TG2) is a peculiar multifunctional enzyme able to catalyze Ca^2+^-dependent post-translational modifications of proteins, by establishing covalent bonds between the peptide-bound glutamine residues and either lysine residues or mono- and poly-amines. In addition, it may also act as a G protein in a transmembrane signaling, and, depending on the interacting partner, as a kinase or a protein disulfide isomerase (PDI). Finally, TG2 can serve as a cell surface adhesion mediator [45,46]. Many studies have shown that TG2 has a role in the major pathways involved in proteostasis maintenance. In fact, it has been recently demonstrated that TG2 is involved in autophagosome maturation and in the post-translational modification of high molecular weight aggregates, which are then conveyed by cargo proteins to the autophagic machinery for degradation [47,48,49]. It has also been shown that TG2 regulates protein homeostasis through exosomes biogenesis by controlling the selectivity of their cargo. Indeed, TG2 influences the recruitment in the exosomes of various proteins involved in proteostasis [50]. Finally, a proteomic analysis of TG2 interactome revealed that the enzyme interacts with various protein categories of which the most represented is a well-defined group of chaperones such as Hsp70 [51]. Our previous study demonstrated that TG2 plays a key upstream role in the regulation of proteostasis by controlling the HSF1/Hsp70 axis. Specifically, TG2, through its PDI activity, catalyzes the trimerization of HSF1, promoting the formation of three intermolecular S-S bonds between two cysteine residues (Cys36 and Cys103), which are essential for HSF1 trimerization and DNA binding (Figure 1) [40,41]. In fact, the absence/inhibition of TG2 impairs translocation of the HSF1 trimeric complex in the nucleus and in turn, the Hsp70 expression [41].

### 2.2. TG2 and HSF1 Axis in the Regulation of the HSR

Many mechanisms regulate the HSF1 activation upon stress conditions. In fact, several stimuli as proteotoxic stress, pathogens and toxins lead to the activation of an inert pool of HSF molecules, in order to stimulate the transcription of stress-responsive genes, which initiates the HSR. HSF protein family consist of different paralogs; however, HSF1 is the main transcription factor involved in the activation of HSR. McMillan et al., first showed that HSF1 deletion impairs HSR responsiveness to acute stress, due to a deficiency in the HSPs expression [52]. Therefore, it is a common knowledge that HSF1 is the king regulator of HSPs expression and the major mediator of their induction in cancer.

**Figure 2:**
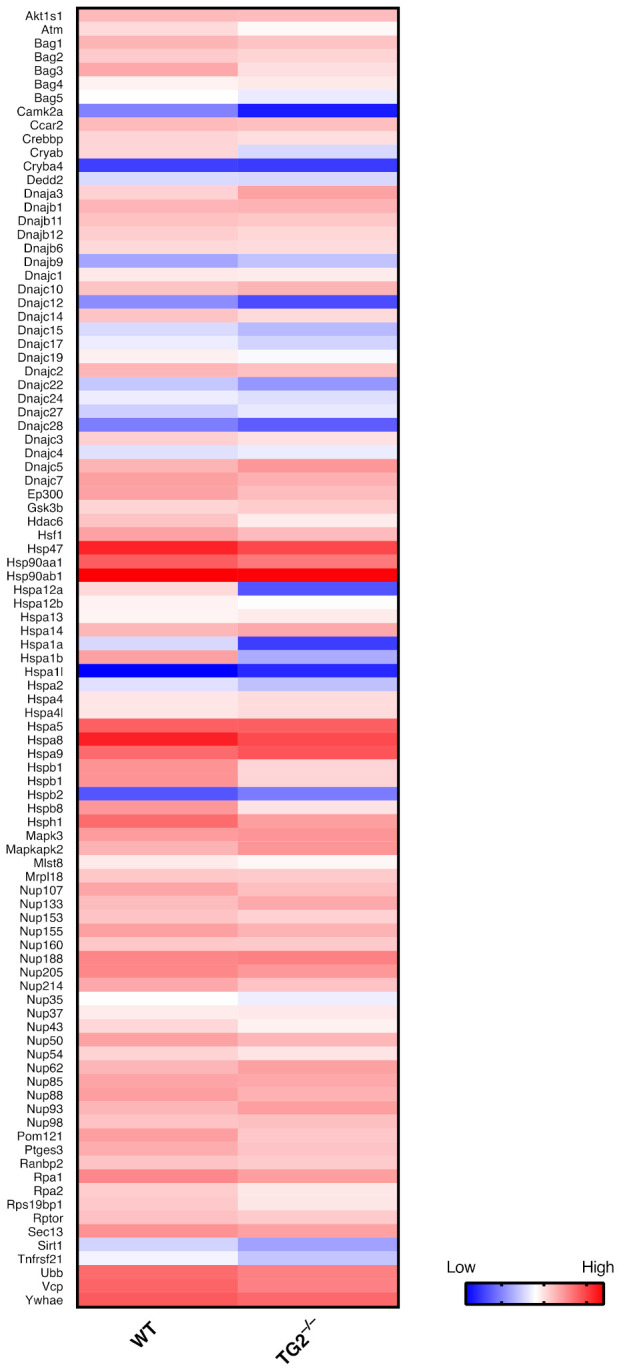
TG2-dependent regulation of HS. Heat map representing the expression of genes associated with the HS response in WT and TG2 knock out cells (TG2^−/−^) after HS induction. Higher expression is red, while lower expression is shown as blue.

As mentioned above, our recently published results suggest that TG2, by promoting HSF1 activation, is a key regulator of the HSR and its function is evolutionary conserved. Indeed, mice lacking TG2 display a markedly impaired response to the HS due to the absence of TG2-dependent HSF1 trimerization. This notion has also been confirmed in human models, where the TG2 inhibition affects HSF1 activation [41]. Interestingly, also in low vertebrates such as *Danio renio* TG2 is essential for a correct induction of the HSR. Specifically, transient knockdown of TG2 in the presence of heat shock (HS), by incubation of larvae at 37 °C, partially but significantly impaired the Hsp70 expression. Accordingly, RNA-seq analysis in wild-type and TG2 knockout mouse models confirmed that the absence of TG2 drastically alters the cellular response to HS. Indeed, in KO cells the number of upregulated or downregulated genes, after HS induction, was significantly lower compared with the WT cells [53]. Interestingly, Gene Set Enrichment Analysis, using both “cellular response to heath stress” and “regulation of HSF1 mediated heat shock response”, reveals many genes downregulated in cells lacking TG2, thus confirming that the enzyme is necessary for a proper response to the HS. Specifically, the expression of many HSPs such as HspA1A, BAG3, DnaJ and many others is impaired in the absence of TG2. In fact, among these impaired genes, HSF1 is also downregulated (Figure 2). These data confirm the previous study indicating that TG2 is necessary for a proper activation of the HS response thus highlighting a specific role for the enzyme in the regulation of HSF1-dependent gene expression.

In this regard, a number of papers highlighted TG2 as a potential regulator of gene expression. Even though TG2 was initially considered a cytosolic protein, it’s now clear that under specific physiological conditions TG2 translocates into the nucleus where it interacts and modifies several proteins [41,54,55,56]. Accordingly, the primary sequence of TG2 contains two putative nuclear localization signals (NLS) and the binding to an importin-α3/Qip-1 family protein may occur to facilitate its transport into the nucleus [57]. Nuclear TG2 is able to modify HSF1, but post-translational modifications and interactions with other transcription factors (e.g., E2F1, Sp1) and histones have been also reported [54,55,56].

Kojima’s group demonstrated that TG2 is responsible for the cross-linking mediated inactivation of the transcription factor Sp1, resulting in the reduced expression of growth factor receptors such as c-Met and consequent hepatocyte apoptosis [56]. Interestingly, it has been reported that HSF1 and Sp1 cooperate for the transcription of some genes including the heat shock protein HspA1B [58,59], thereby corroborating the notion that TG2 could be part of functional nuclear complexes regulating gene expression.

The peculiar biochemistry of TG2, as well as its capacity to interact with the main proteins involved in the regulation of proteostasis imply that TG2, besides its function in the activation of HSF1, directly regulates the HSPs. Indeed, about 40% of TG2 interacting proteins are related to the chaperone protein family [51] and several studies revealed TG2 interactions with many HSPs such as Hsp70, Hsp27, Hsp90 as well as co-chaperones from the DnaJ and the BAG families [60,61,62,63]. Through these interactions, TG2 is able to modulate cellular processes involved in different pathologies. Accordingly, of particular interest is the interaction of TG2 with BAG3 a member of the BAGs family, a group of anti-apoptotic proteins, sharing the BAG domain, that binds and regulates the activity of various HSPs. BAG3 is a co-chaperone involved in the clearance of protein aggregates through the proteasome and/or autophagy and has been reported to form a multichaperone complex with HspB8 and Hsp70. This complex is implicated in the Huntington’s Disease since triggers the selective degradation of mutated huntingtin through a lysosomal degradation process called BAG3-mediated selective macroautophagy [64]. However, it has been reported that when cellular proteostasis is impaired, the clearance of pathological huntingtin could occur via exosomes and is mediated by TG2 interaction with BAG3 [50]. TG2 interaction with HSPs has been proved to occur also in neuronal cell death where the interaction with Hsp20 and Hsp27 plays a protective role against cytotoxic insult [60].

## 3. HSF1 in Diseases

### 3.1. Cystic Fibrosis

Cystic Fibrosis (CF) is an autosomal recessive disease caused by mutations in the gene encoding for the cystic fibrosis transmembrane conductance regulator (CFTR), a 1480 aa cAMP- regulated Cl- channel expressed at the apical membrane of epithelial cells in the airways and in other tissues [65,66]. Currently, about two thousand mutations of the CFTR have been identified, where the most common is the deletion of phenylalanine 508 residue (F508del), turning out in a defective protein folding, that leads to CFTR degradation [67,68]. CF pathogenesis is characterized by imbalance of proteostasis due to an increase in HSF1 trimers and consequently Hsp70 expression, which in turn is involved in F508del CFTR degradation by the proteasome [69]. In this regard, new therapeutic approaches, known as “potentiators” and “correctors”, aim to augment or repair function of the CFTR protein. In particular, the correctors are molecules able to stabilize CFTR, facilitating its folding, minimizing the proteasomal degradation and consequently increasing its stability at the cell membrane [70]. Many sources of evidence suggest a pathogenic role of TG2 in CF since the presence of the mutation F508del in the CFTR induces persistent activation of the enzyme [71,72]. Interestingly, the ablation of TG2 in F508del mice significantly ameliorates the typical CF symptoms improving their survival. In fact, it has been shown that the improvements, observed in the absence of TG2, were paralleled by a reduction in Hsp70 levels indicating that the enzyme regulates the Hsp70 expression also in CF pathogenesis [41]. Recently, it has been demonstrated that the administration of cysteamine, a known inhibitor of TG2, promotes a general amelioration of the disease in CF patients by reducing inflammation and restoring the CFTR function [73,74]. Indeed, the treatment with cysteamine, interfering with the PDI activity of TG2, influences HSF1 trimerization, reducing the amount of the active trimerized form. Moreover, the inhibition of TG2 by cysteamine, leads to a reduction in Hsp70 protein levels as well as in Hsp40 expression, a co-chaperone of Hsp70 required for CFTR F508del degradation. This evidence suggests that TG2, by regulating the HSF1-Hsp70 pathway, could promote F508del CFTR degradation triggering CF pathogenesis. According to this, it is very likely that TG2 inhibition by cysteamine restores CFTR function by affecting the HSF1 trimerization and consequently Hsp70 induction [41].

### 3.2. Neurodegenerative Diseases

**Figure 3:**
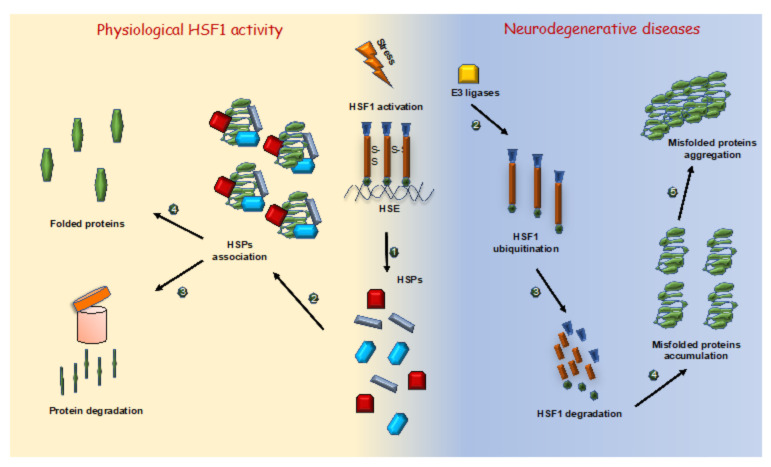
HSF1 action in physiological condition and in neurodegenerative diseases. *Left panel.* During a period of proteotoxic stress, HSF1 trimers bind HSE sequences promoting HSPs expression (1). Misfolded proteins are bound by HSPs (2) driving them toward proteasomal degradation (3) or facilitating their folding (4). *Right panel*. In neurodegenerative diseases several E3 ligases interact with HSF1 (2) promoting its ubiquitination and degradation (3). The reduction in HSPs expression leads to an accumulation of misfolded proteins (4) turning out in the formation of toxic aggregate (5).

Protein aggregation is associated with the onset and pathogenesis of diverse neurodegenerative disorders, such as Alzheimer’s disease (AD), Parkinson’s disease (PD), amyotrophic lateral sclerosis (SLA), dementia with Lewy bodies (LB), and Huntington’s disease (HD) [75]. Accumulating data have supported a potential involvement of TG2 in neurodegenerative diseases [76]. In fact, neurovegetative disorders are characterized by an alteration of proteostasis, in which TG2 plays a key role [42]. Most importantly, numerous studies showed that dysregulation of TG2 may contribute to the pathogenesis of many neurodegenerative disorders, including HD, AD, PD and ALS as well as nervous system injuries [77]. However, the precise mechanism underlying TG2’s role in these disorders remains unclear. In fact, endogenous misfolded proteins can undergo to aggregation, losing their normal function, and can form large insoluble aggregates that are deposited on the tissues, such as the brain and heart, causing organ damage [78]. Since these disorders are characterized by a failure of proteostasis, chaperones play a key role in the pathogenesis of neurodegenerative diseases [79]. Indeed, HSPs partially stabilize the unfolded proteins, dissociate the protein aggregates and drive the misfolded proteins to degradation, making HSF1 the main target factor that needs to be regulated in the neurodegenerative diseases (Figure 3) [80].

It has been shown that the pathological α-synuclein, the common marker of PD and LB, can induce an aberrant degradation of the HSF1 protein, via activation of Nedd4-1, an intracellular E3 ligase, leading to a decrease in the chaperone protein expression [81]. Similarly, in the HD, mutated huntingtin protein (Htt) is able to increase the interaction between HSF1 and Fbxw7, a Skp1-Cull-F box ubiquitin ligase protein complex, through HSF1 phosphorylation on Ser303 and Ser307. This interaction triggers HSF1 degradation [82]. Finally, the expression of HSF1 protein is reduced during AD accompanied with a significant decrease in the expression of the heat shock chaperones including Hsp60, Hsp70 and Hsp90 [83]. Contrary, HSF1 overexpression in the cerebellum has been shown to rescue HSPs expression and mitigate the loss of Purkinje cells [83,84,85,86]. Moreover, overexpression of HSPs has been reported to reduce the number and size of accumulated aggregates and ameliorate the phenotypes in neuronal cells [87]. In addition to endogenous chaperones, a novel class of low molecular weight compounds termed “chemical chaperones” has been shown recently [88]. These molecules contain a hydrophobic core that interacts with non-polar exposed regions of proteins, interfering with hydrophobic packing and disrupting intermolecular hydrogen bonds. Acting similar to “proteostasis-keepers”, these chemical chaperones confer anti-aggregation activity to proteins prone to associate with each other [89]. Taken together, this evidence suggests that both TG2 activity and HSF1 function are essential in many neurodegenerative disorders, thus supporting the close relationship between these two proteins.

### 3.3. Cancer

HSF1 involvement in cancer is a highly discussed topic in the recent years and it is becoming clear that HSF1 supports tumor cell proliferation, survival, invasion and metastasis in a wide range of cancers. Notably, HSF1 activity correlates with a higher rate of survival in several cancer types: breast [90], lung [91], prostate [92], colon [93], myeloma [94], pancreas [95] and hepatocellular carcinoma [96].

**Figure 4:**
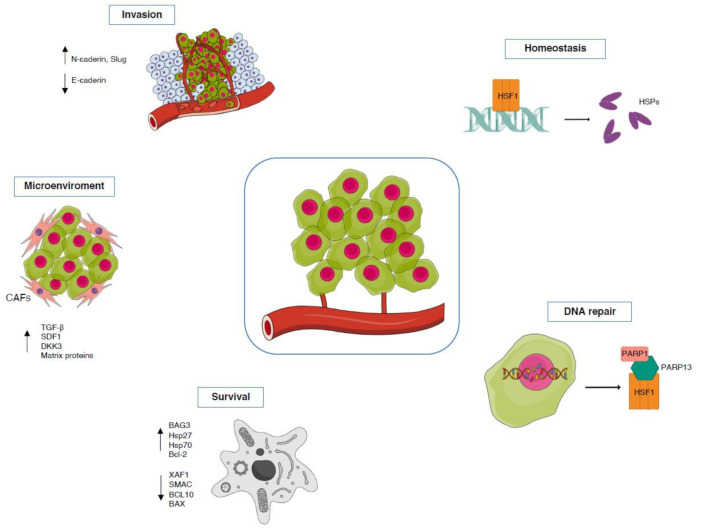
HSF1 multiple roles in cancer development. (Homeostasis) Mutated proteins accumulate in tumor leading to an increase in the HSPs production mediated by HSF1. (DNA repair) HSF1 regulates DNA repair in cancer cells by forming a ternary complex with PARP13 and PARP1 thus favoring PARP1 redistribution to DNA lesions. (Survival) HSF1 promotes cancer cell survival either by inducing the expression of genes encoding pro-survival proteins or repressing genes encoding pro-apoptotic factors. (Microenvironment) HSF1 modulates tumor microenvironment as it is activated in CAFs where regulates signaling pathways involved in ECM remodeling and cancer progression. (Invasion) HSF1 promote EMT either inducing the expression of N-cadherin and mesenchymal markers or downregulating the expression of E-cadherin and epithelial markers.

Functions of HSF1 in tumorigenesis involve the regulation of multiple processes including maintenance of homeostasis, inhibition of apoptosis, control of DNA repair, promotion of tumor invasion and regulation of tumor microenvironment (Figure 4).

Tumors are characterized by the accumulation of mutated and overexpressed proteins that requests an increase in the protein quality control. This increase demand in folding activity induces the HSR, leading to the HSF1-dependent transcription of the molecular chaperones, known to increase cell survival both through direct chaperoning of misfolded proteins as well as inhibition of programmed cell death [97].

Indeed, HSF1 promotes cell survival by inducing the expression of genes encoding pro-survival proteins such as BAG3, Hsp27, Hsp70 and Bcl-2 [98,99,100]. Otherwise, the transcriptional program of HSF1 in cancer, beyond the expression of HSP genes, also involves the repression of genes encoding pro-apoptotic proteins such as XAF1, SMAC, BCL10 and BAX [101,102,103,104]. Accordingly, several studies report that HSF1 knockdown or its inhibition promote a higher rate of apoptosis in different tumor types [105,106].

Genomic instability is one of the most important factors that lead to cancer development and alterations in the DNA repair pathways facilitate the accumulation of genomic alterations contributing to the survival of cancer cells. However, tumors depend on residual DNA repair functions to repair the damage induced by enhanced replication and genotoxic stress [107]. In this regard, it has recently been found that in breast cancer models HSF1 recruits PARP1 through the damage regulator PARP13, thus forming a ternary complex. In response to DNA damage, this complex induces PARP1 activation, dissociation from HSF1–PARP13 and redistribution to DNA lesions promoting the accumulation of DNA repair factors including RAD51 and 53BP1 [108]. In this context, it has also been reported that under genotoxic stress, such as exposure to ionizing radiation, HSF1 deficiency compromises the cell’s ability to arrest cell cycle progression and impairs DNA damage repair by reducing the levels of RAD51 and 53BP1 [109].

Several studies indicate that HSF1 can drive migration and invasion facilitating the malignant transformation and progression of cancer; however, the detailed mechanisms of its pro-metastatic activity are not fully understood. In cancer cells, HSF1 seems to promote epithelial–mesenchymal transition (EMT) either inducing the expression of N-cadherin and mesenchymal markers or downregulating the expression of E-cadherin and epithelial markers [110,111]. HSF1 ability to promote migration and invasion was demonstrated in hepatocellular carcinoma [112], melanoma [113,114], breast [99], ovarian [110] and pancreatic [115] cancers. In agreement with the notion that HSF1 is involved in EMT, the former was shown to confer drug resistance to cancer cells [116]. In breast cancer, Akt mediates the phosphorylation of HSF1, which stimulates the expression of Slug and triggers the EMT [117]. However, not only HSF1 expression promotes EMT, but it also appears that HSF1 is required for TGF-β-dependent signaling in EMT [108,109]. Indeed, in breast cancer cells, TGF-β induces the activation of HSF1-Akt-CyclinD1 pathway through the FAM3C protein, promoting tumor proliferation and migration [118].

On the other hand, Lindquist’s group found that TGF-β is a target gene of HSF1 in the tumor stroma, playing a key role in the transcriptional re-programming of tumor microenvironment [119]. Recently, increasing evidence highlighted a role for HSF1 in the modulation of tumor microenvironment and specifically of cancer-associated fibroblasts (CAFs) in many human tumors such as melanoma, lung, colon, breast, and prostate carcinomas. Indeed, HSF1 is activated in CAFs where regulates at least two central signaling pathways, TGF-β and SDF1, with a transcriptional program completely different from the one used in the adjacent cancer cells [119]. In this regard, it has been recently demonstrated that in colon cancer, stromal HSF1 drives the transcription of genes encoding matrix proteins (FN1, LAMA1), matrix enzymes (MMP7, MMP9), and matrix chaperones (SERPINH1/Hsp47), inducing ECM remodeling and leading to cancer progression [93]. Another effector of HSF1, that modulates the pro-tumorigenic behavior of CAFs, is the recently found Dickkopf-3 protein (DKK3). DKK3 is an HSF1 target gene that promotes aggressive behaviors of CAFs in breast, colorectal and ovarian cancers. It exerts this effect by potentiating YAP/TAZ activity via canonical Wnt signaling [120].

#### TG2-HSF1 Axis in Cancer

TG2 is involved in various mechanisms that contribute to the onset and proliferation of the tumor such as inflammation, cell proliferation, death processes, angiogenesis, metastasis and chemo resistance [121].

The close relationship between inflammation and TG2 has been variously demonstrated in several physiological and pathological conditions, including cancer [122,123,124,125,126]. In particular, the TGM2 gene is regulated by the factor NF-κB [127], which if activated induces the expression of TNFα, IL-1, and IL-6, cytokines known to be potent inducers of TG2 expression [128]. The association between TG2, inflammation and cell survival is also highlighted by the correlation with TGF-β. Interestingly, similar to HSF1, TG2 is required for the activation of TGF-β [129] and this cytokine is necessary for TG2 expression and activity [130,131,132].

TG2 is also involved in the tumor proliferation phase as it alters the mechanisms of cell death and survival [133]. The enzyme has been extensively demonstrated to have a pro-apoptotic role based on its calcium-dependent crosslinking activity that is required for the formation of apoptotic bodies through post-translational modifications of pro-apoptotic proteins [134,135]. However, in tumor context, anti-apoptotic activities of TG2 have been established through mechanisms dependent on cellular localization and on the activation of specific pathways [122], such as NF-κB [136] or retinoblastoma [137].

TG2 is also involved in advanced tumor stages related to increased aggression, such as metastasis or drug resistance processes. Evidence has in fact shown that in the most advanced stages of the tumor there is an over-expression of TG2. In renal cell carcinoma, TG2 is higher in metastatic than in non-metastatic patients [138], a correlation confirmed also in ovarian cancer [139], breast cancer [140] and in the pancreas [141]. Specifically, TG2 supports the adhesion of cancer cells to the extracellular matrix (ECM) through the interaction with both fibronectin and integrins [142]. Furthermore, post-translational modification of ECM proteins by TG2 seems to be a key step for the progression of tumor cells to metastasis, conferring resistance to metalloproteinases and promoting cell–matrix interactions [143,144].

The evidence supports a role for TG2 in the angiogenesisi processes since the enzyme is highly expressed in endothelial cells with positive effects on blood vessel formation. In particular, it has been demonstrated that inhibition of TG2 expression and transamidase activity causes an arrest in angiogenesis mediated by VEGF [145].

All these notions highlight a role for TG2 in the carcinogenesis processes. Thus, considering the interplay between TG2 and HSF1 and their tumorigenic effect, a deep understanding of this axis in the complex and dynamic interplay between the tumor and its surrounding microenvironment could be of great importance to design new therapeutic approaches for cancer therapy.

## 4. HSF1: A Regulator of Development

HSF1 is a highly versatile transcription factor that is involved in many physiological processes, including development, where plays a vital role. Initial observations of HSF1 importance in the developmental processes came from deletion experiments of Hsf gene in *Drosophila melanogaster*. Indeed, flies that are defective for HSF1 arrest the development at the L2-L3 larval stages [146]. Interestingly, genome-wide gene expression studies showed that this effect was not caused by changes in the expression of the HSPs, rather by other HSF1-dependent gene expression patterns [147,148]. The same evidence was found in *C. elegans* models, where deletion of HSF1 ok600 allele leads to premature arrest of development at the larva stage [149]. About vertebrates, mice lacking HSF1 possess multiple defects such as chorioallantoic placenta, prenatal lethality, growth retardation, female infertility and absence of the HSR; however, they can survive to adult age [147].

Female sterility caused by HSF1 deficit was found both in *Drosophila* and mice [146,150,151]. Indeed, it is becoming clear that HSF1 is essential for oocyte meiosis and, when absent, these cells arrest the meiotic maturation at phase I or II [150,152]. Recently, it has been shown that HSF1 is abundantly expressed in maturing oocytes and its ablation leads to the downregulation of Hsp90. Similarly, Hsp90 inhibitors cause the same phenotype, i.e., the HSF1 deficit [150]. Moreover, oocytes lacking HSF1 present dysfunctional mitochondria and are more sensitive to oxidative stress, showing a reduced rate of survival [153].

HSF1 is a key regulator in the brain development maintaining central nervous system (CNS) proteostasis. Indeed, the absence of HSF1 results in brain morphological alterations with lateral ventricles markedly enlarged, white matter reduced and areas of neurodegeneration [154]. Moreover, brains from HSF1knockout mice exhibit higher levels of ubiquitinated proteins, increased levels of protein oxidation, and sensitivity to oxidative stress, indicating that HSF1 is essential for the maintenance of CNS homeostasis [155]. Another study showed that HSF1 knockout mice present aberrant affective behavior, with depression-like and aggressive features [156]. The analysis of the molecular mechanism revealed that HSF1 directly controls the expression of the polysialyltransferases in the hippocampus, thus modulating PSA-NCAM (polysialylated-neural cell adhesion molecule) levels, known to participate in the remodeling of neuronal circuits [156]. Other genes such as Syt1, Vamp2, Dp71 and LIF1, all involved in neuronal development, have been identified as target of HSF1 further strengthening the evidence for a protective function of HSF1 in brain development [157,158,159,160].

Interestingly, it has recently been demonstrated that HSF1 promotes the activation of the Wnt/β-catenin signaling, a key pathway required for embryonal development [161,162]. Our lately published work suggests that TG2, by regulating HSF1, controls the Wnt signaling and this axis is essential for the correct embryonal development of lower vertebrates [53]. Indeed, TG2 ablation in zebrafish determines a severe developmental impairment starting from gastrulation stage with around 40% of morphants died. Of note, TG2 knockdown represses not only the HSR but also the Wnt pathway leading to progressive alterations in morphology and body shape, thus highlighting the role of the TG2-HSF1-Wnt axis in the developmental processes.

## 5. Conclusions

The HSF1 protein regulates the heat shock response pathway by acting as the major transcription factor for the heat shock proteins. HSF1 is regulated at multiple levels by different signals and proteins, which modulate its activity and function under normal and stress conditions. In this regard, TG2 has now emerged as a key regulator of HSF1 activation, since it is required for HSF1 trimerization and for a proper HSR induction. In recent years, it has become clear that HSF1 not only activates the classical HSP genes during stress, but it also stimulates different transcription programs involved in many physiological and pathological processes. Currently, HSF1 is recognized as a major player in several diseases including cancer and neurodegenerative disorders. Of note, TG2 has also been implicated in these pathologies making the HSF1-TG2 axis an attractive topic to be further exploited from the therapeutic point of view. Indeed, aberrant TG2 activity is found in some neurodegenerative diseases, such as AD, PD and HD [163]. Moreover, this enzyme is involved in the insurgence and progression of several tumors including breast, prostate, renal, pancreatic and liver cancers [138,164,165,166,167]. In this context, additional studies to evaluate HSF1-TG2 interplay would critically contribute to improve the current knowledge on the processes regulated by HSF1 in diseases.

## Data Availability

No data availability.
